# Everybody Can Dance—Except Aging Professional Dancers! A Discussion of the Construction of the Aging Dancing Body in Four Dance Texts

**DOI:** 10.3389/fspor.2022.819572

**Published:** 2022-03-25

**Authors:** Hilde Rustad, Gunn Helene Engelsrud

**Affiliations:** ^1^Kristiania University College, Oslo, Norway; ^2^Western Norway University of Applied Sciences, Sogndal, Norway

**Keywords:** bodily resonance, interaffectivity, phenomenology, document analysis, Butoh dance

## Abstract

The subject of the article is a critical investigation of research concerning age and dance. Our objective is to investigate whether and how researchers express their ideas about dance and age in a selection of research papers. We are particularly interested in whether researchers are reproducing an instrumental understanding of age in the context of dance and whether discourses of dance define bodies as older or younger in ways that differ from the definitions used in other social contexts. What kind of assumptions about the abilities of dancers form the baseline expectations of researchers? We wonder if harming the body is an implicit part of dance practice that operates as a tacit premise in the understanding of age and dance. Through a document analysis of several research texts on dance and age, we try to identify what kinds of meanings, expectations, and bodies such documents convey and produce. One of our findings from the analysis of the literature is that young dancers from western European countries and the U.S. are concerned with age throughout their entire career, while in dance practices in Japan, being an older dancer is regarded as a as a value that gives flavor and energy to both to aging and dance in a shared interaffective and mutual space.

## Background And Research Context

Research on the relationship between age and dance is a growing field, indicated by an increasing number of available publications on the topic. Bolwell (2017, p. 314) states that ageism in dance has existed for decades, and refers to Marcia Siegel who addressed this topic, connected to ballet, already in 1976. However, researchers such as Hansen and Kenny (2019, p. 24) claim that research on “age and dance” is limited. In this article we try to unpack some of the research positions that have been influential in creating a conversation about dance and aging and the aging body in dance, and in doing so we hope to address some of the “limitations“ that Hansen and Kenny refer to. We use “dance” as an encompassing concept and we position ourselves in a process of being and becoming older chronologically speaking and facing society's expectations about our dancing practices and how we experience, feel, perceive, and relate to the concept of being an “aging dancer.”

Being older, white, Norwegian dancing women and dance researchers—and one of us a professional dancer—we (the authors) have personal experience with the ways people understand the relationship between dance and aging. We frequently get questions and comments such as ”Are you still dancing?” “But you don't look that old,” and “Are your hips still functioning?” The cultural assumption seems to be that as their age begins to limit their movement performance, professional dancers should retire. Further, that performance as a category imply a specific (physical) function apart from one's own feeling and kinesthetic awareness and present energy. When exposed to the comments we often feel “stuck” within conventional categories and language, which in the situation can be difficult to respond to. German dancer and researcher Susanne Martin describes asking herself when she was still quite young, “am I already too old to dance?” (Martin, 2017, p. 163). Her question is a common one among professional dancers, many of whom question their performance abilities throughout their careers. Already being “too old” became an embodied experience for Martin from a young age, and her future as a professional dancer seemed in doubt, or at the very least destined to be short lived. Like many of her colleagues in Western dance culture, Martin felt limited by the fact that most dancers stop performing after the age of 40. Due to this cultural practice, not only are dancers deprived of a professional career, but the audience is also deprived of the opportunity to follow dance artists over a longer period of time, and to see the evolution of the body as it bears witness to the aging process through the medium of dance. Audiences over the age of 40 can hardly identify with the young bodies on stage, which in turn reinforces the cultural mismatch between older people and dance. It also means that younger dancers have few role models who can outline long career paths that point to the future (Rustad, 2017, p. 37/13). As Helen Thomas so clearly writes, biological reality is at the heart of the chronology of aging, “Age is something we do, and we cannot stop doing it at will” (Thomas, 2013, p. 108). Thomas refers to Laz who argues that age is “constituted in interaction” and that it gains its meaning in the context of “larger social forces” (Thomas, 2013, p. 108). From this perspective, we cannot help but perform our own age all the time. In such circumstances, it seems almost inevitable that researchers would—perhaps unintentionally—reproduce ideas about aging and dance as antithetical to each other.

There is an emergent paradox in the relationship between aging and dance. On the one hand, we have established that the number of professional dancers above a certain age is limited. On the other hand, another set of cultural expectations and research indicates that, for non-professionals, dancing is good for older people and can improve their health. Dancing gets older people out of their homes, it is an enjoyable way for them to exercise and socialize, and in general it serves to increase quality of life (Krekula et al., 2017). Dance is further shown to have significant health effects for older people as it may improve age affected bodily functions such as balance, sensory motor and cognitive performance (Hokkanen et al., 2008; Kattenstroth et al., 2010, 2013; Ridder et al., 2013). In this context, dance “is used” as a tool to promote and sustain good health and wellbeing, to keep the body moveable and in shape, and to prevent old people from becoming a burden to society. It is tempting to ask whether dancing is good for everybody except professional dancers!

## The Social Construction of Being “Old”

“Old” is a discursive and cultural phenomenon with diverse meanings in various contexts and cultures. However, age functions as a social marker that defines when a person is or becomes “old.” The United Nations defines an older person to be “a person who is over 60 years of age,” but qualifies this definition by stating that other socio-cultural factors can also contribute to defining age, including “family status (grandparents), physical appearance, or age-related health conditions” (United Nations, n.d.). Age of retirement is a fair indicator of what is considered to be old, and it varies accordingly from country to country. In China, the average age of retirement (from a job in the public sector) is 55 for women and 60 for men (China Retirement Age, 2021). In Japan the average retirement age for men is 65 (Japan Retirement Age, 2021).

Norway has a flexible retirement age that ranges from 62 to 75; the average retirement age is 67 (Norway Retirement Age, 2021). An official Norwegian report on artists and their lives (Heian et al., 2015) finds that most dancers end their careers before they become 40. However, it is becoming more common in Norway for dancers older than 50 and 60 to continue working and performing as professionals. To our knowledge most of these older dancers work within contemporary dance, in contrast to classical ballet dancers at the Norwegian National Opera & Ballet, who retire at 41 (The Norwegian National Ballet, n.d.).

Although it may seem difficult to be an older dancer working in a western country and competing for jobs with younger dancers, the situation for older dancers in Norway is quite different than in other countries thanks to the various state subsidies that support artists in Norway. All of the older Norwegian dancers we know of who are still dancing receive economic support from long term state artist scholarships or “The alliance for actors and dancers” (SKUDA, 2021). This suggests that only dancers who get regular financial support are able to continue dancing when they become older. Further, Norwegian professional dancers of all ages may take morning classes for free at PRODA (professional dance training). In Oslo—where most Norwegian dancers live—such classes are offered daily, with the exception of weekends and holidays, and in other parts of Norway weekly or in periods. PRODA is financed by the Norwegian state (PRODA, n.d.).

## Age as Embodied Experience

In understanding age as an embodied social and phenomenological phenomenon, we are inspired by philosopher Maurice Merleau-Ponty (1908–1961), who writes that “our own body is in the world as the heart is in the organism: it keeps the visible spectacle always alive; it breathes life into it and sustains it inwardly, and with it forms a system.” (Merleau-Ponty, 1989, p. 203). The body and the world are dialectically interwoven, through time and the process of aging. When the world changes, everybody in the world changes as well, including dancers. Dancers understand their bodies in relation to culturally institutionalized values. In the west, bodies are often valued for being young, white, thin, and strong. However, these values are derived from externalized discourses that exclude a full, sensuous awareness. In the moment of performance, the shape and visual appearance of young dancers may not seem so different from those of older dancers, in the resonance between their bodies, the Other and the world. Fuchs and Koch (2014, p. 3) define *bodily resonance* as something “that include all kinds of local or general bodily sensations: feelings of warmth or coldness, tickling or shivering, pain, tension or relaxation, constriction or expansion, sinking, tumbling or lifting, etc.” Bodily resonance corresponds both to autonomous inaction and muscular activation. Both old people and small children can feel in their bodies whether the people around them are friendly, irritable, or hostile by the way they move and speak—by their energy, tone, and manner (Cohen, 2018). This type of bodily resonance guides and colors communication, allowing the perceiving body to live and interact with others. Fuchs (2016) links his description of bodily resonance to Merleau-Ponty's (1968) notion of intercorporeality. Fuchs argues that *intercorporeality and interaffectivity* in the close encounters between people who interact with each other can be experienced as a web of bodily resonance processes, characterized by people's mutual incorporation of each other (p. 196). Fuchs emphasizes the mutual bodily resonance that arises in the dynamic interaction of the encounter with others and argues that this resonance is the basis for a common understanding of each other and the world (Fuchs, 2016). The researchers we have mentioned here have inspired us to embark on our own investigation of the literature on age and dance, using both cultural practices and older dancers' bodily experiences as our points of reference.

## Method

In this article we have chosen to analyse documents and “Document analyses” is defined in a variety of ways. In line with Asdal and Reinertsen (2020, p. 43) we have chosen to regard the document as active and possessing textual agency. This means that we believe the texts we read produce knowledge about and perspectives on “age and dance” which might (re)produce cultural ideas related to those phenomena. As Asdal and Reinertsen (2020, p. 43) write, “s*omething happens* both with documents and in documents—but what?” Documents are places for understanding and producing meaning. Age *affects* both dance and dancers, and the documents we read and write establish the contexts and relationships between the dancers and the dance—they tell us *how* and *why*. Documents are put into circulation and movement; they work together and on each other and they shape and reshape their subjects (Asdal and Reinertsen, 2020, p. 16–17). Together, a body of interrelated documents produces a field of research. It is important for researchers to recognize the role that they and the documents they write play in establishing, modifying, and contextualizing their chosen topics. The documents we have analyzed and refer to throughout this article have contributed to the creation of a field of research on dance and aging which they, as existing documents, are also a part of. We are aware that our own process of reading, analyzing and writing about these documents is in turn a part of this growing body of research, and that it may become a living document for future researchers to read and use. As researchers we enter a field of knowledge where age and dance are already being negotiated and contested—the context is evolving, but many of the rules and customs are already established. Both the documents and our analysis of them can influence and change the understanding of “age and dance” as we went our way through established truths and arguments, testing them to see if they still are sound, perhaps breaking them down and rebuilding them with additional materials to form a new perspective.

## Selection of Documents

In selecting which documents to analyze and which to omit, we are actively modifying the research field. Each new document helps to form the topic that it itself investigates (Asdal and Reinertsen, 2020, p. 112–113). The main documents that we have chosen for analysis were published between 2005 and 2017. By looking at how research documents on age and dance build upon each other—or do not—we have the ability to trace how documents bring the past with them (Asdal and Reinertsen, 2022, p. 135).

Three of the four main documents we analyze in this article are written by researchers who have done in-depth research on dance and age: Elisabeth Schwaiger, Nanako Nakajima, and Susanne Martin. All three have published books on age and dance [Schwaiger's book is *Aging, Gender, Embodiment and Dance; Finding a balance* (2012), Nanako Nakajima and Gabriele Brandstetter's book is titled *The aging body in dance* (2017) and Susanne Martin's *is Dancing Age(ing); Rethinking Age(ing) in and through improvisation Practice and Performance* (2017)]. Our fourth main document is written by dancer, filmmaker, and choreographer Yvonne Rainer.

We have chosen documents where the authors investigate various dance practices, and our intention is to see what we find when we bring the documents together and make them communicate with each other. Although we include references to amateur and community dance, we have focused our research mainly on professional theater dance, selecting shorter texts whose titles suggest or clearly communicate age and dance as their themes.

For professional dancers, getting older usually has a strongly negative impact on their careers from an age that would still be considered relatively young in most other professions. Being overly concerned about how you look on stage undermines the value of expression, communication, identification and interaffectivity based on mutually shared spaces and experiences. Schwaiger argues that the world of dance is particularly hard on the old, saying that “Western culture does not, as a rule, value old age, and nowhere is this more salient than in careers such as professional dance, which, like professional sport, gymnastics or modeling, is clearly a body-based domain” (Schwaiger, 2012, p. 1). In 1988 Judith Lynne Hanna wrote that when it comes to age, dance is “unique among the performing arts,” because older dancers face an increased risk of physical injuries. Hanna characterizes dancers' careers as short-lived because older bodies are unable to meet up with the strenuous demands placed upon them (Hanna, 1988, p. 121). Hanna claims that age is one of the primary reasons why professional dancers end their careers. However, researchers such as Albright (1997) and Østern (2015, p. 5) problematizes the stereotype of young, slim, strong bodies as the cultural norm, and Østern argues that they obscure all other body types from view and create a perception of dance as an artform enacted solely by thin, flexible young bodies in good physical shape (Østern, 2015, p. 5, in Rustad, 2017, p. 6). In other words, professional dancers who fit this stereotype reproduce the idea that dance is an artform performed only by young people.

### Four Main Documents, Chronologically Listed

Schwaiger, L. (2005). Performing one's age; cultural constructions of aging and embodiment in western theatrical dancers. *Dance Res. J*. 37, 107–120. 10.1017/S014976770000838X

Nakajima, N. (2011). De-aging dancerism? The aging body in contemporary and community dance. *Perform. Res*. 16, 100–104. 10.1080/13528165.2011.606033

Rainer, Y. (2017). “The Aching Body in Dance.” In: Nakajima, Nanako and Brandstetter, Gabriele (eds.) *The Aging Body in Dance*, p. 31–34.

Martin, S. (2017). *Dancing Age(ing); Rethinking Age(ing) in and through improvisation Practice and Performance*. Blelefeld: Trancript publishing.

### Liz (Elizabeth) Schwaiger

Shwaiger's article “Performing One's Age: Cultural Constructions of Aging and Embodiment in Western Theatrical Dancers” was published in 2005, in Dance Research Journal, and is part of her Ph.D., thesis. Schwaiger lives in Western Australia. She begins her article with two citations exemplifying what she calls contrasting approaches to age and dance in western countries:

*For the dancer, [the] aging process is a painful and difficult one. It is especially so in our Western culture with its focus always on youth and its image of the dancer being one…, of agelessness, of continually having a brilliant technique and youthful agility. Therefore, after about the age of thirty-five years, the dancer has to confront not only the natural thresholds of age but also the social pressures of our culture, in particular the pressures within the dance culture*. (Cameron-Dalman, 1996, 33, in Schwaiger, 2005, p. 107, author's italics).

This passage addresses the way the image of young dancers' *brilliant technique and youthful agility* is deeply perceived as both a *natural threshold* and *a social pressure of the dance culture*. She writes about the aging process as a painful one that becomes embodied for most dancers by the *age of 35 years*. This corresponds with what Berson (2010) writes about “dance culture” when she points out that “even those of us who study and participate in intergenerational dance have been trained to make aesthetic judgements based on technical virtuosity, which privileges and norms the abilities of youthful bodies rather than the engagement and the experience of aged bodies” (Berson, 2010, p. 175). However, how older dancers experience dancing when becoming older might be quite different:

*When you are young, you go in all directions, you can do everything, you are expanding, you like to expand, you like to fill spaces with your energy. But then, and this is what is so nice when you get older, you start this process of reducing, of reducing what you would like to say … and this is fantastic*. (Quote from 55-year-old female dance artist, as part of Schwaiger's Ph.D., work, in Schwaiger, 2005, p. 107, + Note 1, p. 118, author's italics).

Schwaiger's article is based on interviews with 21 female and nine male dancers aged 40+, conducted in Australia, Germany and Ireland. According to her, the reasons to not dance when becoming older have to do with “the specific historical, cultural, and social expectations of the performing dancer's physicality in its maturity [that] construct and mark it as aged” (Schwaiger, 2005, p. 108). Similarly, Berson (2010) writes that when older dancers perform on stage, it may reify degrading myths about old age. She mentions the tendency to think of older bodies as being in a process of decay (Berson, 2010, p. 165), and writes that “most concert dance tends to reify dominant notions about the body, including the aging body …” (Berson, 2010, p. 168).

Schwaiger (2005, p. 108) asks whether we are “compelled to act our age,” and points out that a “middle-aged female dancer is expected to not only dance differently from a young dancer, but also be costumed differently from her, to mark her as middle-aged.” She questions why there are so few older dancers and posits that it is because society's expectations negate the experience of the older dancers. However, she argues that postmodern dance practices, as opposed to classical dance and ballet, make it quite possible for dancers to continue dancing “well into older age” (Schwaiger, 2005, p. 109)

Interestingly, Schwaiger (2005) claims that there are significant differences between Australia and America, arguing that Australia lacks postmodern dance and accordingly, “ballet—as esthetics and a system of values and training—remains the standard for all Western theatrical dance and what is known as “contemporary dance [in Australia]” (109). We believe this to be true for other countries as well. Indeed, most Europeans associate dance with ballet and are not able to differentiate between dance genres. This also means that many people have a vague, generalized understanding of aging dancers and believe that all kinds of professional dancers should and do retire early.

Within the tradition of classical ballet, age-related physical decline is expected to stop dancers from doing physically demanding movement work. However, within postmodern and today's contemporary dance, things are different. Schwaiger refers to Sally Gardener, who in her master's thesis from 1997 wrote about a dancing subject and subjectivity in flux, showing how different dance genres produce different dancing subjects, and here Gardener builds upon Susan Leigh Foster's book Reading Dancing from 1986 (Schwaiger, 2005, p. 110). Schwaiger writes that Gardner's understanding is that when it comes to postmodern dance subjectivity is redefined, and “movement is already encultured in the body” and it was about attempting to “allow the body a significance of its own” (Schwaiger, 2005, p. 111).

What this means in relation to aging dancers is an opportunity to continue to practice and perform beyond the supposed glass ceiling of their mid-thirties or early forties, indeed for the rest of their active lives, since this paradigm of subversion of ageist readings of bodies allows for normative constructions of aging to be challenged (Schwaiger, 2005, p. 111).

By referring to Gardener, Foster, and others, and in this way bringing former research into her document, we get a sense of how Schwaiger created her case, and her references help us as readers and researchers to expand the scope of research on dance and age.

### Yvonne Rainer

“The Aching Body in dance,” written by choreographer and dancer Yvonne Rainer, was first published in 2014 when Rainer was 80 years old and republished in 2017. In her article Rainer writes about the modern history of dance, famous choreographers and dancers, and she brings her own personal history into the account as she remembers watching old dance artists perform when she herself was quite young. When Rainer was young she watched Ruth St. Denis performing at 78 (“The flabby undersides of her upper arms created their own autonomous swaying motion” p. 3), Isadora Duncan's foster daughter Maria-Theresa (“I was impressed that she could still run” p. 3), Martha Graham (“later she could be embarrassing as she tried to inhabit the roles that she had created for herself in her younger days” p. 3). Rainer's choice of words might be interpreted as a young dancer's view and description of older dancers in performance. As Rainer herself was a dancer, it is interesting that there seems to have been little contact between her and the older dancers she describes. Hansen and Kenny (2019, p. 24) claim that “scholarly research and published artistic reflections reveal negatively charged terminology used by aging dancers in the Western world to describe their reduction of movement range, their loss of professional opportunities and the representation of the aging body on stage.” In other words, Rainer and dancers like her show little understanding or sympathy for their own aging bodies or those of other dancers. Rainer mentions dancers closer to herself in age, such as Merce Cunningham, Trisha Brown and Paul Taylor, but without making critical, negatively charged age-related remarks.

After having retired from dance at the age of 40, Rainer returned in the year 2000, age 66, and toward the end of the article she describes how she “at present” deals with performing as an older dancer. Although Rainer was quite critical of old choreographer-dancers' performances when she herself was much younger, she ends her article by encouraging herself now as an old dancer to keep on dancing. She writes “Farewell to mewling ‘I no longer dance.' Dance girl, dance, and to all who observe me, I challenge you, ‘Pity me not.”' (Rainer, 2017, p. 6). Rainer's answer to her question “So when is it time to say, ‘farewell to dance?”' (Rainer, 2017, p. 3) seems to be an emphatic “never!” However, this last quote is specifically addressed to herself, and does not necessarily encourage others to keep performing after becoming old. We interpret Rainer's text to be written from the perspective of her dancing body—a subject full of the desire to continue dancing.

Rainer argues that one of the ways that the shift toward postmodern dance in the 1960s, in which she was involved, changed dance was by introducing pedestrian movements to its vocabulary. She writes that the “evolution of the aging body fulfills the earliest aspirations” of postmodern dance (Rainer, 2017, p. 6) and argues that “it is high time to admit the aging body of the dancer into this by now fully recognized and respected universe” (Rainer, 2017, p. 6). It is easy for Rainer to claim, in retrospect, that the values associated with postmodern dance encourage the participation of older bodies. However, Nakajima (2011, p. 100) addresses this matter specifically and points out that the performers at Judson Church Dance Theater were fit and healthy, and that the work was “based on a logic tied to a young, able body.” Although, today one might read about the shift to postmodernism in dance and interpret its new more democratic values as inclusive of a variety of bodies, including older bodies, this does not seem to have been the case.

### Nakaijima, Butoh, and Bodily Memory

In her document “De-aging Dancerism? The aging body in contemporary and community dance” (2011) Nanako Nakajima connects age with themes such as postmodern dance, community dance, and Japanese Butoh dance.

Franko (2011) has also published research on Butoh, and both Franko and Nakajima write about Japanese Butoh-dancer Kazuo Ohno (1906–2010). Franko describes Ohno's performance *Admiring La Argentine*, in which Ohno performs his “memory image” of the Spanish flamenco dancer Antonia Mercé (1890–1936) who used “La Argentine” as her stage name, and whom Ohno saw dance in person in 1929 (Franko, 2011, p. 106). Sixty years later, the first author of this article, Rustad, saw Kazuo Ohno perform *Admiring La Argentina*; at the Stadsschouwburg, a theater in Amsterdam. At that time Ohno was 83 years old and celebrated as a world-famous dance artist and innovator who was given recognition for being central in the initiation and development of Butoh. The Stadsschouwburg was packed with people, and after having concluded his performance Ohno came back on stage again and again as his audience kept on applauding, and the applause appeared to be endless. The way he repeatedly danced his way on and off stage, somehow became part of the performance itself. Rustad wrote in her notes from the performance that “his graceful movements seemed to me exotic, unusual, inspired and beautiful. He seemed fragile, and with strong intention. Was deeply affected.” Rustad's memory of the tiny old man dressed and made up as a woman, still touches her and resonates in her body. She likes to think Ohno, while performing, experienced bodily resonance with his memory impression of ‘La Argentine's” dance. There is a line of interconnectedness that extends from the present memory back to Ohno's performance in 1989, which in turn was a response to a dance performance from 1929. This can be described as a web of bodily resonance processes (Fuchs and Koch, 2014) that stretches across space and time, spanning decades and continents.

When Ohno performed in the Stadsschouwburg, the audience was enthusiastic and treated him with respect and admiration. The audience's response contrasted with the way that western society usually sees and treats old people, and more specifically the way western society treats older professional dancers. In fact, in Norway where we live and work, we cannot remember having witnessed any performance with a professional 83-year-old Norwegian dancer in it.

The culturally based Western understanding of aging is largely a negative one. Getting older is regarded as unhealthy and as a process that diminishes wellbeing and makes life darker and more difficult for people in general, and especially people within the field of dance. According to Friedrich Nietzche, western culture generally favors visual qualities over kinesthetic experience. Nietzche believed that the kinesthetic and the tactile are suppressed by the visual and auditory senses. The visual and auditory are partners with the intellect, while the kinesthetic and tactile follow and are followed by movement and bodily engagement, listening and human knowing (Berg Eriksen, 1989).

Nakajima writes that “when dancers become old and lose control over their bodies, their bodies lead and their minds follow” (Nakajima, 2011, p. 102) This again relates to Nietzche's ideas about the kinesthetic and experienced—and points further to the intercorporeal dimension, through which audience members experience dance and dancers not only with their eyes and ears, but through their entire body as an integrated part of the performance. When intercorporeality occurs, the audience become social participants, instead of distant observers.

Nakajima describes a Japanese approach toward dance and age that is quite different from the one in the West. She says that, in Japan, people believe that professional dancers become better performers as they get older, and that in contemporary Butoh, “aging causes no degeneration of dance forms, but it accelerates a dancer's development of the artistic faculty” (Nakajima, 2011, p. 102). In traditional Japanese dance “aging is thus the ultimate status of dancing for those professional dancers, and the audience wants to spend money to watch them dancing” (Nakajima, 2011, p. 103). Nakajima describes dance as a lifelong practice of artists as craftsmen and uses the examples of Ohno and Anna Halprin, successful older dancers from Japan and the U.S., both in contrast to the dominant trend in the west, what she calls “the negative prejudice against aging bodies in Western dance” (Nakajima, 2011, p. 102). Nakajima argues that this prejudice reduces the market value of older dancers in the U.S.

According to Nakajima, Ohno does not trust Modern dance technique as he claims it transforms him into nothing more than a technician. She writes that Ohno's knowledge of dance is bodily integrated, allowing him to forget about it so that he can achieve freedom in his dancing (Nakajima, 2011, p. 103). Further, Franko writes that Ohno's “aging body increasingly became the subject and the theatrical wager upon which his work was based” (Franko, 2011, p. 125). In other words, far from being a hindrance, the aging process can actually be a source of inspiration for dance art, a raison-d'être for the performance.

Even when taking into consideration the differences between Japanese and western societies, it clearly seems easier being a dance artist and have a lifelong career in Japanese society. Fraleigh (2016, p. 64) writes that neither perfect bodies nor mastery and perfection are valued in Butoh, and the Japanese understanding of age and dance shown in Nakajima's article—exemplified through Ohno—is in stark contrast to the Norwegian and European understanding of age and dance, especially as it is manifested in the world of ballet. In Japan, dancers are not excluded from performing due to old age. Older Westerner dancers have taken note, questioning why older dancers are seen so differently in the East. Rosita Boisseau has interviewed Western dancers who are still performing past the age of 70, among them 72-year-old American Carolyn Carlson. Boisseau writes:

These performers don't believe in a best-before date. They dismiss the notion of dance as the preserve of youthful, technically impressive virtuosos. This is a “very western” view, according to Carlson, who is influenced by Buddhism and eastern spirituality and cites the “national treasures” of live performance in Japan and South Korea. (Boisseau, 2016, p. 2).

### Suzanne Martin

Suzanne Martin is a dancer and dance researcher who in her book *Dancing Age(ing); Rethinking Age(ing) in and through improvisation Practice and Performance*—has written what she calls an “age autobiography”^1^:

*My psychological age is flexible and shifting, but since I started to become a dancer, I often felt much older than I looked. I see this self-perception as related to three circumstances related to the field of professional dance. Firstly, having danced throughout my entire childhood and youth I have a very long and rich practice history, and I will soon celebrate my fortieth stage anniversary. This produces a kind of veteran self-image. Secondly, I went through an ‘age-socialization (Gulette*, *2004**, p. 12) of being always already too old, or soon too old for dance. Thirdly, since my mid-twentieth I regularly experience my body as failing, as vulnerable, unpredictable, painful, exhausted*. (Martin, 2017, p. 62, author's italics)

Martin underlines that she was already worried about the presumably short lifespan of her career as a dancer when she was still quite young. She saw getting older as an obstacle to dance, and her perception of the aging body was established from a young age. She lived with these ideas, and they formed an embodied barrier that seemed to limit her ability to perceive herself, to take joy in dancing and being a dancer.

Foster says that professional dancers must constantly “apprehend the discrepancy between what they want to do and what they can do.” As they get older, they find it increasingly difficult to respond to new performance projects, not to mention “the devastating evidence of aging” (Foster, 2003, p. 237). Dancers are constantly living with the pressure to be better, stronger, more graceful, and yet condemned to know that the mastery they hope to achieve will always be out of reach. Even as dancers are training to be more skilled, they are looking in the mirror for signs of aging, which are taken to indicate that they are “slipping,” and can no longer perform at the highest levels (Rustad, 2017, p. 5).

The premium placed on youth in dance is a reflection of the status of youth in society at large. Jowitt (1994) writes that “onstage, as in life, the ultimate compliment is “You do not look your age.” Nevertheless, Hansen & Kenny found that older dancers they interviewed had an “advanced understanding of their dancing bodies, which they have actively pursued or learned from lived experiences” and that, out of necessity, these older dancers were able to “adapt and modify for their aging bodies” despite the internal pressure of perfectionism, and external pressures such as choreographer and audience expectations (Hansen and Kenny, 2019, p. 25–26).

## The Aging Body in the Context of Being a Professional Dancer: Toward an Ending

The chosen method of document analysis highlights some aspects and exclude others. We acknowledge that there are limitations to our claims in this paper as we are considering no more than four texts. The content of our chosen texts reveals experiences of dancers of different genders and age groups, who belong to quite different cultures,—and precisely how cultural differences influence dancers could have been interesting to dive more into. However, it has not been the purpose and scope of this research project to cover these factors in depths.

In our analysis of the selected texts, we have discovered cultural variation that, from a phenomenological perspective, favors older dancers who treat aging as a privilege that allows them and their audience to participate in a shared space where both can feel that “the body is in the world as the heart is in the body.” In this sense the body and the world are dialectically interwoven, not limited by age or aging. However, many dancers become aware of their body in relation to a cultural gaze that seeks to define older bodies as out of place. However, these values are derived from externalized discourses that give short shrift to touch and bodily resonance. Whatever the public perception, an older body still resonates within itself, and between itself, the Other and the world. Inspired by Thomas Fuchs and Sabine Koch's definition of bodily resonance (Fuchs and Koch, 2014, p. 3), we believe that dancers and audience experience both autonomous inaction and muscular activation, in a shared sensory field of bodily resonance that is characterized by mutual incorporation (p. 196). As in Ohno's performance attended by the first author, mutual bodily resonance arises in the dynamic interaction of the encounter between audience and performer. This resonance is the basis for a shared understanding of each other and the world. The dancer, with his sensitizing body, attuned the audience and vice versa. De Jaegher et al. (2017) operationalize the phenomenon of intersubjectivity as follows:

In order to make intersubjectivity “graspable” and to operationalize its investigation, we need characteristics of social action that are concrete and “handy” to be accessible to experience and testable in a research setting. For this, we employ three characteristics of social perception: its spatiality, its sociality, and its modalities of sensing, feeling, and thinking (De Jaegher et al., 2017, p. 499).

Demographically speaking, most western people can expect to become rather old, and because getting old is something most of us hope to achieve it is paradoxical that, in certain contexts such as dance, old people and old bodies are made undesirable, even invisible in western society. At the same time, old dancers are praised in other parts of the world. In those cultures, coming together through dance in an intersubjective and interaffective context can help to bridge age gaps and generate intercorporeal experiences.

There are other good reasons for older dancers to keep on dancing as well, and researchers have shown how dance improvisation allows bodies of all ages to unfold themselves in performance. Martin writes “I recognized that dedicated specialist practitioners of improvisation in their fifties and older share an approach to work and life that is continuously open to possibility and in contradiction to the culturally dominant age(ing) narratives” (Martin, 2017, p. 163). Nakajima describes how Kazuo Ohno forgets, but dances from his bodily memory, and that this gives him freedom in dance. Rainer writes that she loves to exist on stage, and that, at 80 years old, she can give herself roles other than that of a dancer (such as reader of text) (Rainer, 2017, p. 5–6). Hansen and Kenny believe that more professional dancers will be able to continue their work into old age if they are able to “balance pressures with the ability to listen to their bodies” (Nakajima, 2019, p. 27).

We have shown, using document analysis, that Western dance produces and reproduces cultural expectations about aging and older dancers. This causes young dancers to worry about becoming older when they are still young and makes it difficult for them to pursue lifetime careers as performing artists. On the other hand, Japanese dance, as exemplified by Kazuo Ohno's performance, shows how older dancers can add value to dance, and how, in itself, the experience of aging may become a source of inspiration for dance art.

By analyzing and comparing several different perspectives on the relationship between dance and aging, our article may contribute to modifying and changing how people consider this theme. Expectations about the normal age of dancers in both East and West are influential in their respective societies. We believe that the main documents we have chosen to investigate, as well as the other authors we have referred to in this article, show that it is difficult for western performers to contravene deeply established ideas about dance and age. Across the various documents, our analysis shows that being affected and affecting others is a universal phenomenon that occurs in social spaces where people interact around the world. The experience of watching Kazuo Ohno's performance shows how the interaffective aspect of performance operates independent of the performers' or the audience member's age.

Yet, the published research on age and dance also reveals some of the barriers that prevent western dancers from continuing their professional careers into old age. The idea that the aging process is biologically predetermined is dominant in the west. People in the world of European and American dance believe it is simply not possible for older dancers to perform demanding dance movements with the skill or ability of younger dancers. Because they suffer by comparison, it is considered better for the older dancers to stop dancing altogether. Furthermore, the social norms of retirement strongly encourage professional ballet dancers to stop dancing/retire around the age of 40. Most people seem to think that older people should not be dancing on stage—even within the context of contemporary dance. Living within static concept understandings and cultural construction in society, it is not surprising that most professional dancers (and choreographers) accept or abide by the public opinion that there is no place for older people on stage. Injuries from performance or training also cut short the careers of many older dancers. Another factor is economy. Due to competition for jobs, it is difficult for dancers to get paid work even when they are young—and it certainly doesn't get any easier when they are older.

Analysis of existing literature (Coupland, 2013; Southcott and Joseph, 2019) indicate that, although there is potential for older dancers in western countries to bring a unique and powerful expression to dance, getting old generally prevents dancers from performing. Age is not considered a valuable resource in western dance culture. Dance performances that derive their power from the dancers' maturity, experience, and ability to dig deeply into the subject of the performance are not to be found. Older audience members are not given the opportunities to share social space with performers who are their peers; instead, they are consigned to being spectators, watching young dancers whose skills they might admire, but do not identify with. The invisibility of older bodies on stage shapes the expectations of the audience as well as the dancers, and over time it limits the expressive power of the artform. Showcasing older dancers more often on stage and increasing the number of performances by dancers of different ages makes good sense, especially given the research that shows the benefits that dancing has on health and wellbeing. It is a pity if the only people who cannot enjoy dancing into their old age are professional dancers.

## Data Availability Statement

The original contributions presented in the study are included in the article/supplementary material, further inquiries can be directed to the corresponding author/s.

## Author Contributions

HR and GE contributed to conception, design of the study, and have been collaborating throughout the process of writing the article. Both authors contributed to manuscript revision, read, and approved the submitted version.

## Conflict of Interest

The authors declare that the research was conducted in the absence of any commercial or financial relationships that could be construed as a potential conflict of interest.

## Publisher's Note

All claims expressed in this article are solely those of the authors and do not necessarily represent those of their affiliated organizations, or those of the publisher, the editors and the reviewers. Any product that may be evaluated in this article, or claim that may be made by its manufacturer, is not guaranteed or endorsed by the publisher.

## Footnotes

^1^‘Age autobiography' is referred to as a concept developed by Gulette (2004).
